# Effects of iRoot SP on osteogenic differentiation of human stem cells from apical papilla

**DOI:** 10.1186/s12903-021-01769-9

**Published:** 2021-08-18

**Authors:** Laidi Wu, Kaiyang Xue, Guang Hu, Hanman Du, Kang Gan, Juanfang Zhu, Tianfeng Du

**Affiliations:** Department of Stomatology, The First Affiliated Hospital of Zhengzhou University, No.1 Jianshe East Road, Zhengzhou, 450052 Henan China

**Keywords:** Nano-bioceramics, iRoot SP, Human stem cells from apical papilla, Osteogenic differentiation

## Abstract

**Background:**

Research shows that nano-bioceramics can modulate the differentiation of dental stem cells. The novel ready-to-use calcium-silicate-based root-canal sealer iRoot SP is widely used in root filling. Accordingly, the aim of this study was to evaluate the effects of iRoot SP on proliferation and osteogenic differentiation in human stem cells from the apical papilla (hSCAPs).

**Methods:**

hSCAPs were isolated and characterized in vitro, then cultured with various concentrations of iRoot SP extract. Cell proliferation was assessed by CCK-8 assay, and scratch-wound-healing assays were performed to evaluate cell-migration capacity. hSCAPs were then cultured in osteogenic medium supplemented with iRoot SP extracts. Alkaline phosphatase (ALP) activity assay was used to evaluate ALP enzyme levels. Alizarin red staining and cetylpyridinium chloride (CPC) assays were performed to assess calcified-nodule formation and matrix-calcium accumulation of hSCAPs. The mRNA and protein expression levels of the osteogenic markers OCN, OSX, Runx2, and DSPP were determined by qRT-PCR and Western blotting. The data were analyzed using one-way ANOVA and LSD-t tests.

**Results:**

iRoot SP at low concentrations (2, 0.2, and 0.02 mg/mL) is nontoxic to hSCAPs. iRoot SP at concentrations of 0.02 and 0.2 mg/mL significantly increases cell-migration capacity. In terms of osteogenic differentiation, 0.2 mg/mL iRoot SP promotes intracellular ALP activity and the formation of mineralized nodules. Moreover, the expression of osteogenic markers at the mRNA and protein levels are upregulated by iRoot SP.

**Conclusion:**

iRoot SP is an effective filling material for periapical bone regeneration.

**Supplementary Information:**

The online version contains supplementary material available at 10.1186/s12903-021-01769-9.

## Background

Pulpal and periapical diseases are common endodontic conditions, and root canal therapy (RCT) is currently the most effective treatment for such conditions [1]. However, previous studies have revealed treatment-failure rates of 4–15%, even after refined RCT treatment, with such failures evolving into refractory apical periodontitis characterized by persistent inflammatory response and progressive destruction of periapical bone, eventually resulting in tooth loss [2, 3]. Accordingly, increasing numbers of studies are being focused on developing new clinical strategies for the repair and/or regeneration of damaged endodontic tissue.

Mesenchymal stem cells (MSCs) are often used to repair inflammatory tissue damage owing to their multi-differentiation ability and anti-inflammatory properties [4, 5]. Human stem cells from the apical papilla (hSCAPs) are a type of dental mesenchymal stem cells first reported by Sonoyama et al. [6]. They exist in the undeveloped apical area of young permanent teeth and exhibit superior self-renewal, colony formation, cell migration, and multi-differentiation compared to other dental stem cells [7]. Accordingly, in vivo studies have confirmed that hSCAPs present remarkable tissue-regeneration capabilities [8]. Furthermore, under inflammatory conditions, they maintain stemness and exhibit better osteogenic differentiation than that observed under normal conditions [9]. Consequently, hSCAPs show great promise for use in the regeneration of dentin-bone-like tissues after pulp and periapical disease treatment as well as having potential clinical value as seed cells in bone-tissue regeneration [10, 11].

Bioceramic materials based on calcium silicate have been widely developed for tissue repair and regeneration [12, 13]. One such material, iRoot SP, is a recently developed ready-to-use nano-bioceramic material that is mainly used for root canal sealing and filling. iRoot SP is an aluminum-free injectable paste composed of calcium silicate, calcium phosphate, calcium hydroxide, zirconia, and fillers [14]. Recent studies have indicated that appropriate concentrations of mineral trioxide aggregate (MTA), the current gold-standard repair material, promote the expression of mineralization-related genes (BSP, Runx2, DSP, and OCN) in hSCAPs by activating MAPKs cascade signaling pathways [15], and that iRoot SP has comparable or superior biocompatibility and osteogenic potential to those of MTA [16, 17]. Furthermore, studies on the interactions between filling materials and cells have revealed that iRoot SP promotes osteogenic differentiation in MG63 cells, human tooth germ stem cells, and human periodontal ligament stem cells, confirming its excellent biocompatibility and mineralization effect when used for root filling [18–20]; while a previous study by our group showed that iRoot SP has a persistent killing effect on *Enterococcus faecalis*, which is closely related to refractory periapical inflammation [21]. Nevertheless, the biological effects of iRoot SP on hSCAPs have not been reported.

Accordingly, the purpose of the current study was to determine whether iRoot SP affects the proliferation, migration, and osteogenic differentiation of hSCAPs and to provide an experimental basis for further research into the molecular mechanisms of interactions between iRoot SP and hSCAPs.

## Methods

### Isolation and culture of hSCAPs

Normal impacted third molars with immature roots were obtained from 10 healthy subjects aged 12–18 years. Apical papilla was separated from the root apex of the extracted tooth. After isolation, the tissues were washed with phosphate‐buffered saline (PBS), minced, and digested in a solution of 3 mg/mL collagenase I (Sigma-Aldrich, St. Louis, MO, USA) and 4 mg/mL dispase II (Roche Diagnostics Corp., Indianapolis, IN, USA) for 30 min at 37 °C. Then, the cell suspensions were centrifuged at 1000 rpm for 5 min, resuspended, and seeded into 25 cm^2^ culture flasks. The cells were cultured in complete medium (α-MEM medium supplemented with 10% fetal bovine serum, 100 μg/mL streptomycin, and 100 U/mL penicillin) (Gibco, Grand Island, NY, USA). These cells were defined as P0 and incubated in a humidified atmosphere of 5% CO_2_ at 37 °C. The medium was changed every 2 days. When grown to approximately 80% confluence, the adherent cells were detached with 0.25% trypsin–EDTA solution (Sigma) for 1 min at 37 °C and then passaged at a ratio of 1:2. Cells in the 3rd to 5th passages were used in this study.

### Flow cytometry analysis

To confirm the identity of the hSCAPs, flow cytometry was used to analyze the expression of stem cell surface markers. Briefly, hSCAPs were trypsinized and resuspended in PBS, then the cell density was adjusted to 1 × 10^7^ cells/mL. hSCAPs were incubated with fluorescence-conjugated monoclonal antibodies (CD45-FITC, CD24-APC, and CD146-PE; Biolegend, San Diego, CA, USA) in the dark at room temperature for 20 min. Their isotype control antibodies were used to determine nonspecific fluorescence. After rinsing twice with PBS, the cells were subjected to analysis on a flow cytometer (BD Biosciences, San Jose, CA, USA).

### Immunofluorescence

hSCAPs were seeded on glass coverslips at a density of 1.0 × 10^4^ cells/well. After incubation for 24 h, the cells were fixed using 4% paraformaldehyde solution and then permeabilized with 0.5% Triton X-100 for 15 min at room temperature. The permeabilized cells were blocked with 1% bovine serum albumin for 1 h and then incubated with primary antibodies for CD24, STRO-1, vimentin (Abcam, Cambridge, UK), and keratin (Cell Signaling Technology, Danvers, MA, USA) overnight at 4 ℃ in the dark. The cells were then incubated with the specified fluorescence-conjugated secondary antibody (Invitrogen, Carlsbad, CA, USA). Finally, 4',6-diamidino-2-phenylindole (DAPI) mounting medium was added to the samples and images were acquired using an inverted fluorescence microscope.

### Osteogenic differentiation

To investigate the in vitro osteogenic-differentiation ability of hSCAPs, cells were seeded onto six-well plates (2 × 10^5^ cells/well) and incubated in complete culture medium. Once grown to ~ 60% confluence, the medium was replaced with osteogenic medium to which 50 μg/mL ascorbic acid, 10 mmol/L β-glycerophosphate, and 10^˗8^ mol/L dexamethasone (Sigma) were added. After 3 weeks of induction, cells were fixed in 2% paraformaldehyde and stained with 1% Alizarin red (Sigma) at room temperature for 30 min. The calcified nodules were photographed using a phase contrast microscope.

### Material preparation

Under sterile conditions, the iRoot SP (Innovative BioCreamix Inc, Vancouver, BC, Canada) was prepared according to the manufacturers’ instructions and placed into plastic Eppendorf microcentrifuge tube lids. Each individual sample was covered with a wet cotton pellet and placed in an incubator under 5% CO_2_ at 37 °C for 3 days. The solidified materials were then removed from the lids and ground into powders. After disinfection with ultraviolet light for 1 h, they were immersed in 10 mL α-MEM medium for 3 days in an incubator to allow the soluble components to leach into the medium. The extracts were then collected and filtered using a 0.22-μm sterile filter. Subsequently, the iRoot SP extracts were diluted to the desired final concentrations with complete medium and stored at 4 °C before use.

### Cell viability

hSCAPs were seeded in 96-well plates at a density of 3 × 10^3^ cells/well. After being cultured in complete medium for 24 h, the medium was replaced with different concentrations of iRoot SP extract and cocultured for another 1, 3, or 5 days. Cell viability was determined using CCK-8 assays. Briefly, the hSCAPs were rinsed with PBS and then 10% CCK-8 solution (Dojindo, Kumamoto, Japan) was added to each well before incubation for 2 h at 37 ℃ under 5% CO_2_. Then, the optical density at 450 nm was measured using a microplate reader.

### Cell migration

hSCAPs (5 × 10^4^ cells /well) were seeded into six-well plates in complete medium until 80% confluency was reached. A 200 μl sterile pipette tip was used to make a central scratch through the monolayer of cells. Cell debris were rinsed away with PBS and the samples were incubated with various concentrations of iRoot SP extract for up to 12 h to allow cell migration back into the wound area. The wound areas were measured at 0 and 12 h using a phase contrast microscope equipped with a digital camera. The wound widths were quantified using ImageJ software (National Institute of Health, Bethesda, MD, USA).

### Alkaline phosphatase (ALP) activity assay

To assess the effect of iRoot SP on the osteogenic potential of hSCAPs, the cells were seeded in 12-well plates at a density of 1 × 10^4^ cells/well and incubated in complete medium. Once grown to ~ 60% confluence, the cells were cocultured with osteogenic medium containing different concentrations of iRoot SP extract for 3, 7, or 14 days. Then, the cells were lysed in Western and IP cell lysates (Beyotime Biotechnological Inc., Shanghai, China) for 20 min. ALP activity in the lysate was determined using an ALP activity detection kit (Beyotime) following manufacturer’s instruction. The absorbance at 405 nm was measured with an ELISA microplate reader. To normalize the activity of ALP, protein concentrations were determined using BCA kits (Beyotime). According to the results of the above experiments, 0.2 mg/mL iRoot SP was used for further study.

### Alizarin red staining

After osteogenic induction of hSCAPs treated with 0.2 mg/mL iRoot SP for 3, 7, 14, or 21 days, the cells were rinsed with PBS and fixed with 2% paraformaldehyde, then stained using 1% Alizarin red (Sigma) for 30 min at room temperature. The stained calcified nodules were observed using a phase contrast microscope. For quantification, the stained nodules were dissolved in 10% cetylpyridinium chloride (CPC) monohydrate solution (Sigma) for 20 min and the supernatant was pipetted into a 96-well plate, and the OD at 562 nm was measured using a microplate reader.

### qRT-PCR assay

To determine the expression levels of osteoblast‐related genes in iRoot SP‐treated SCAPs, the total RNA was extracted from each group of cells using trizol reagent (Invitrogen) at 3 and 7 days. The purity and concentration of the RNA was assessed using a Nanodrop 2000 (Thermo, Wilmington, DE, USA). Finally, the RNA was dissolved in RNase-free water and stored at − 80 ℃ until use. The extracted total RNA was then reverse-transcribed into complementary DNA using a RevertAid First Strand cDNA Synthesis Kit (Applied Biosystems, Carlsbad, CA, USA) according to the manufacturer’s instructions. The cDNA obtained was then used as a template for PCR. The specific primer sequences used for qRT-PCR are listed in Table 1. GAPDH was used as a housekeeping gene for normalizing the expression level of mRNA. qRT-PCR was performed with a QuantiTect SYBR Green PCR kit (Qiangen, Hilden, Germany) on an ABI RT-PCR detection system (Applied Biosystems). The amplification conditions were 95 °C for 10 min followed by 40 cycles of 95 °C for 15 s and 60 °C for 60 s. Each sample was analyzed in triplicate. The data were analyzed using the ΔΔCt method.

**Table 1:** Primer sequences used in qRT-PCR

Genes		Primer sequences (5′–3′)
OCN	Forward	AGGGCAGCGAGGTAGTGAAG
	Reverse	CCTCCTGAAAGCCGATGTGG
OSX	Forward	CCAGGCAACACTCCTACTCCA
	Reverse	GCCTTGGGTTTATAGACATCTTGG
Runx2	Forward	CTACTATGGCACTTCGTCAGGAT
	Reverse	ATCAGCGTCAACACCATCATT
DSPP	Forward	ACAGTAGTAATAACAGCAAGGGCC
	Reverse	CACTGCTGGGACCCTTGATTT
GAPDH	Forward	GGAAGCTTGTCATCAATGGAAATC
	Reverse	TGATGACCCTTTTGGCTCCC

### Western blot

Cells cultured under the same osteogenic-induction conditions were lysed in RIPA lysis buffer (Beyotime) after 3 and 7 days. Protein concentrations were measured with BCA protein assay reagent. Equal amounts of protein for each sample were separated using 12% sodium dodecyl sulfate–polyacrylamide gel electrophoresis and then transferred onto poly(vinylidene fluoride) membranes. The membranes were then blocked with 5% nonfat milk and incubated with primary antibodies for OCN, OSX Runx2 (Affinity Biosciences, Cincinnati, OH, USA), and DSPP (Santa Cruz Biotechnology Inc, Santa Cruz, CA, USA) at 4 °C overnight and then incubated with horseradish-peroxidase-conjugated secondary antibody (Proteintech, Wuhan, Hubei, China) for 1 h at room temperature. Immunoreactive bands were visualized using an enhanced chemiluminescence kit (Thermo Scientific) and the grayscale values were determined with ImageJ software.

### Statistical analysis

Statistical analysis was performed using SPSS 21.0 software. All experiments were conducted in triplicate, and the data are expressed as mean ± standard deviation. Group comparisons were performed via one-way analysis of variance (ANOVA) followed by least significant difference testing. Differences were considered statistically significant at *P* < 0.05.

## Results

### Characterization of hSCAPs

**Fig. 1:**
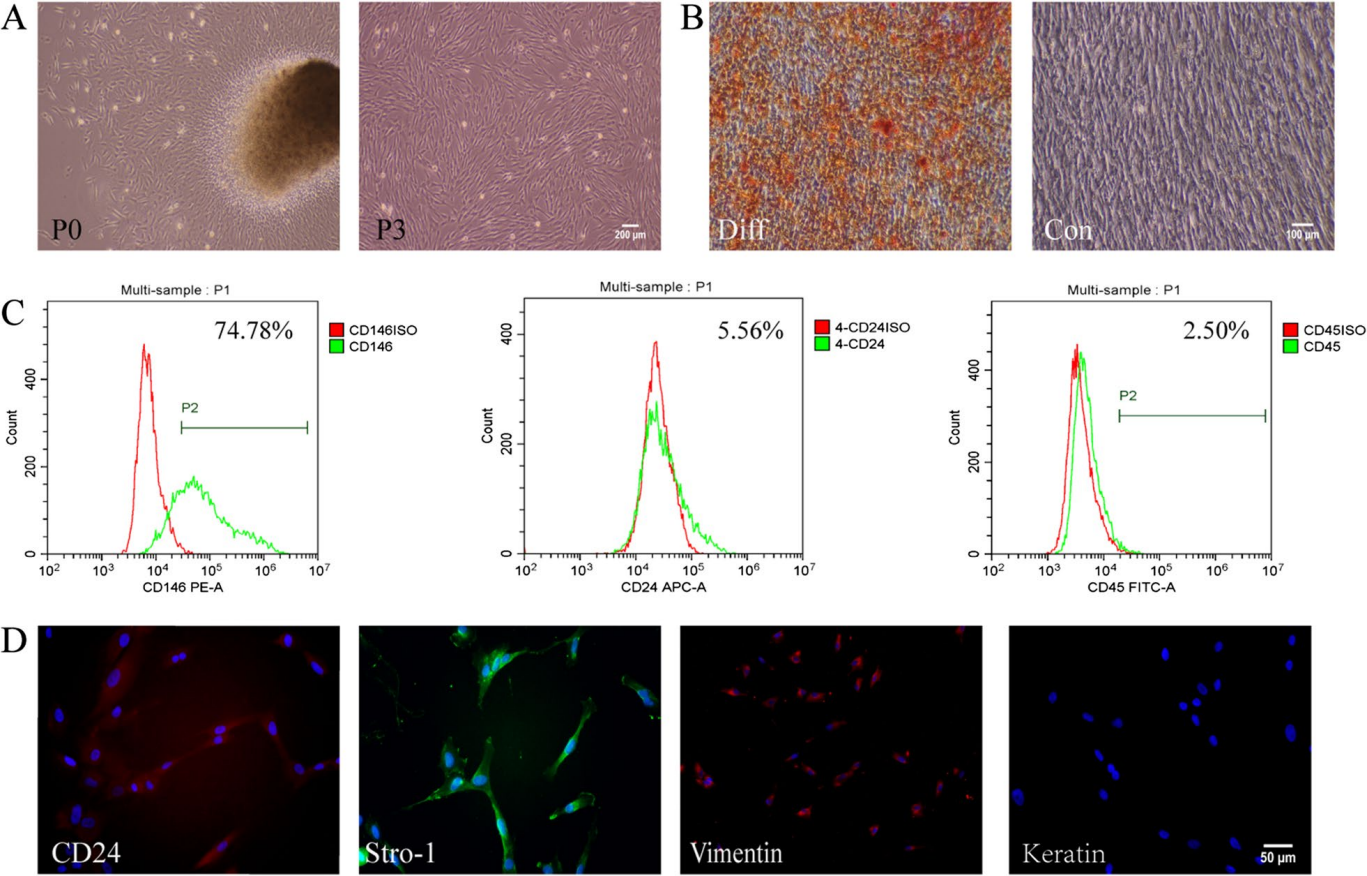
Isolation, culture, and characterization of human stem cells of the apical papilla (hSCAPs). **A** Primary cultured hSCAPs at 7 days (P0). The 3rd passage hSCAPs exhibit spindle-like morphology (P3). **B** Osteogenic differentiation of hSCAPs: hSCAPs were cultured with or without osteogenic-induction factors for 3 weeks, the mineralized nodules formed are positive to Alizarin red staining. **C** Flow cytometry analysis of hSCAP surface markers. hSCAPs test positive the for mesenchymal stem cell markers CD24 and CD146 and the negative expression hematopoietic stem cell marker CD45. **D** Characterization of hSCAPs by immunofluorescence staining: positive for vimentin, STRO-1, and CD24, but not for keratin

Primary hSCAPs were isolated from the apical papilla tissues by enzyme digestion and presented typical cell colonies at approximately 7 days. The 3rd passage hSCAPs were observed to be spindle-shaped (Fig. 1A). Upon culturing in osteogenic-differentiation medium for 3 weeks, hSCAPs form mineral nodules, which are positively stained with Alizarin Red S (Fig. 1B). Flow cytometry indicated that the cells express MSC surface markers, including CD146 and CD24, but not the hematopoietic marker CD45 (Fig. 1C). Immunofluorescence staining of the hSCAPs revealed that the cells positively express CD24, STRO-1, and vimentin, but not keratin (Fig. 1D). Thus, our obtained cells were confirmed to be MSCs.

### Effects of iRoot SP on the cell viability of hSCAPs

**Fig. 2:**
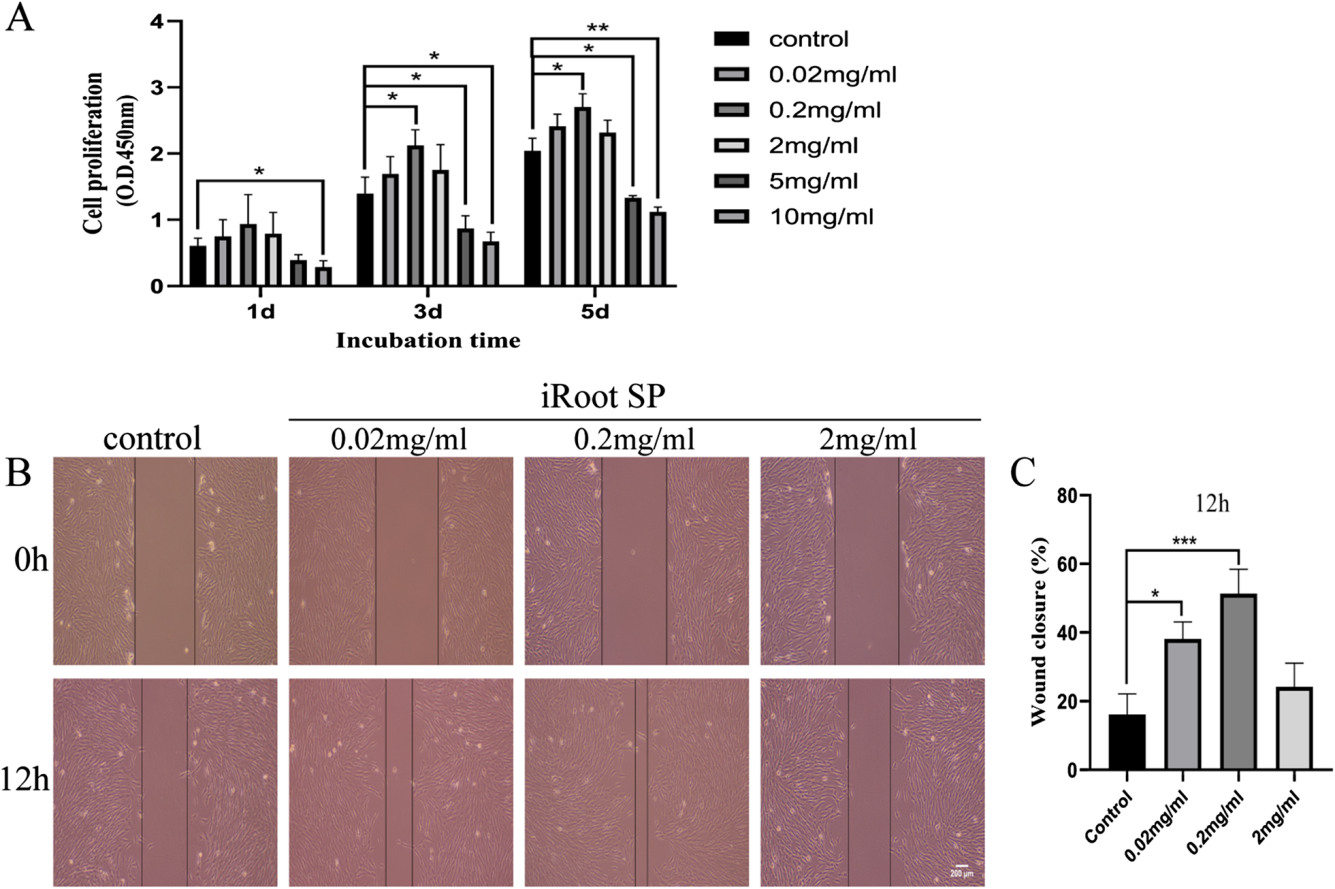
Cell proliferation and migration capacity of hSCAPs cultured with iRoot SP extract at various concentrations. **A** hSCAP proliferation was measured by CCK-8 assay. The results show that 0.2 mg/mL extract improves the proliferation level at 3 and 5 days compared with the control, and that there is no significant difference between the 0.02 mg/mL, 2 mg/mL, and control groups. **B** Cell migration processes as observed under a microscope. **C** Wound-closure percentages of hSCAPs cultured in iRoot SP at 12 h. Scratch-wound-healing assays show that the migration capacity of hSCAPs cultured in 0.02 and 0.2 mg/mL iRoot SP extract were significantly increased compared with the control group. *P < 0.05, **P < 0.01, and ***P < 0.005. Error bars: mean ± standard deviation

CCK-8 assays were used to measure the viability of hSCAPs cultured in media with different concentrations of iRoot SP. The variation in cell viability between all the groups at 1 day is not significantly different (*P* > 0.05). However, the proliferation of hSCAPs measured at 3 and 5 days is significantly higher for the group exposed to iRoot SP at 0.2 mg/mL as compared with those for the other groups (*P* < 0.05). Furthermore, there is no significant difference between the 2 mg/mL, 0.02 mg/mL, and control groups in terms of cell viability. However, higher concentrations of iRoot SP (5 and 10 mg/mL) clearly inhibit cell proliferation over the period studied (*P* < 0.05) (Fig. 2A).

### hSCAPs migration

The effect of iRoot SP on the migration of hSCAPs was evaluated by wound-healing assays. The wound closures for hSCAPs cultured with 0.02 and 0.2 mg/mL iRoot SP extract are significantly increased at 12 h as compared with that of the control group (*P* < 0.05). However, hSCAPs incubated in 2 mg/mL iRoot SP extract exhibit no significant difference in cell migration compared with the control group (*P* > 0.05) (Fig. 2B, C). Thus, the results show that iRoot SP is biocompatible and has beneficial effects on hSCAPs at appropriate concentrations.

### Effects of iRoot SP on the osteogenic differentiation in hSCAPs

**Fig. 3:**
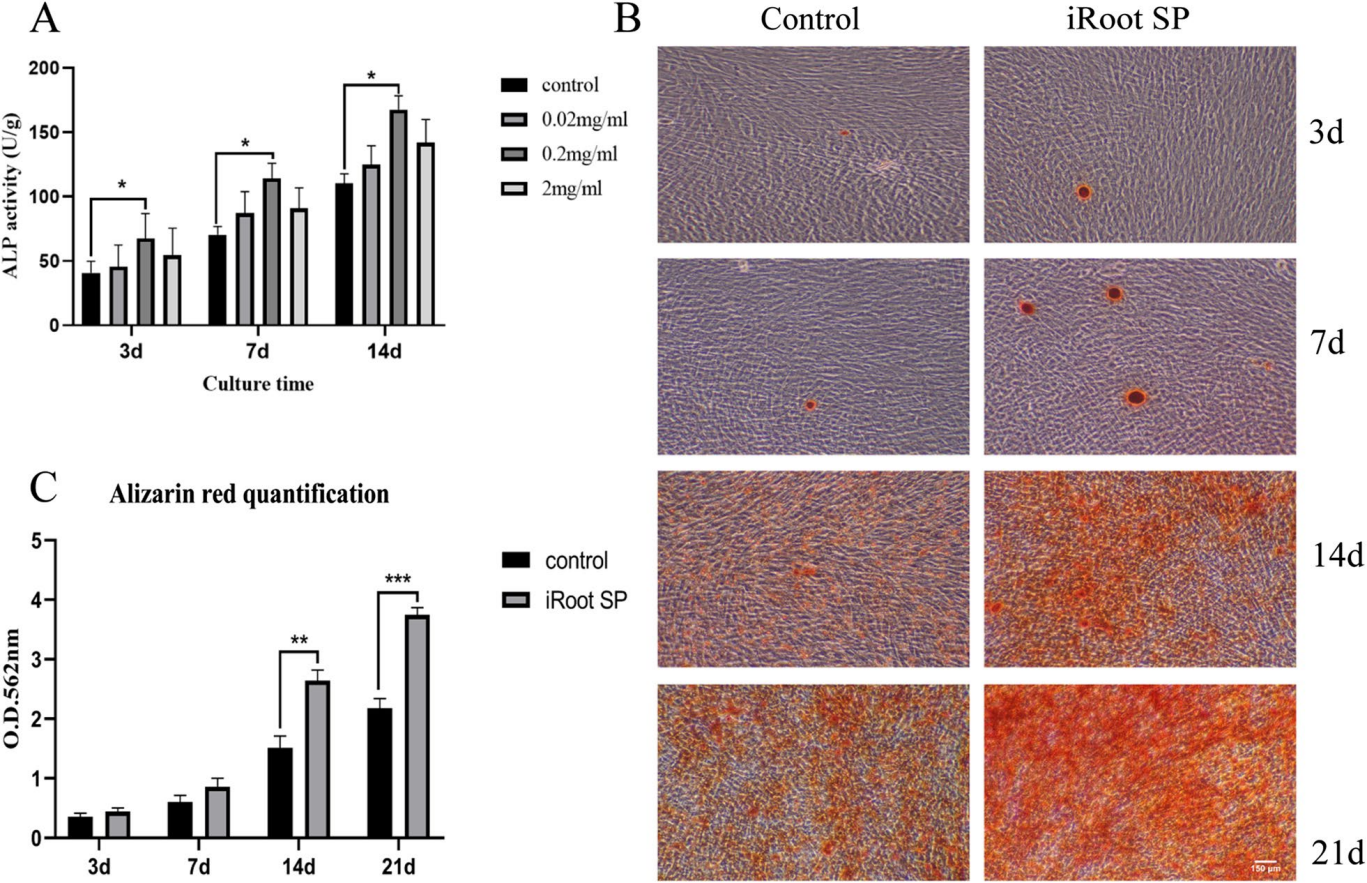
Effect of iRoot SP on ALP and mineralization for hSCAPs. **A** ALP activity was assessed on days 3, 7, and 14. There is no significant difference in the ALP levels between the 2 mg/mL, 0.02 mg/mL, and control groups. The 0.2 mg/mL group presents the highest ALP activity. **B** Alizarin red staining shows that 0.2 mg/mL iRoot-SP-treated cells generate more mineralized nodules than those subjected to the control treatment. **C** The calcium contents of the iRoot-SP-treated cells are significantly higher than those of the control cells (as determined by CPC assay). *P < 0.05, **P < 0.01, and ***P < 0.005. Error bars: mean ± standard deviation

As indicated by the results of ALP activity, at 0.2 mg/mL, iRoot SP extract clearly upregulates ALP activity as compared with that in the control group (*P* < 0.05, Fig. 3A) at different time points. Additionally, there are no statistical differences between the 2 mg/mL, 0.02 mg/mL, and control groups over the period studied. Furthermore, Alizarin red staining and CPC assays demonstrated that hSCAPs exposed to iRoot SP extract at 0.2 mg/mL generate more mineralized nodules and present higher calcium contents compared with the control group after 14 and 21 days (*P* < 0.01) (Fig. 3B, C), but no significant effects are observed at 3 and 7 days. Therefore, 0.2 mg/mL was selected to be the optimal concentration to measure the differentiation capacity of SCAPs in the following experiments.

**Fig. 4:**
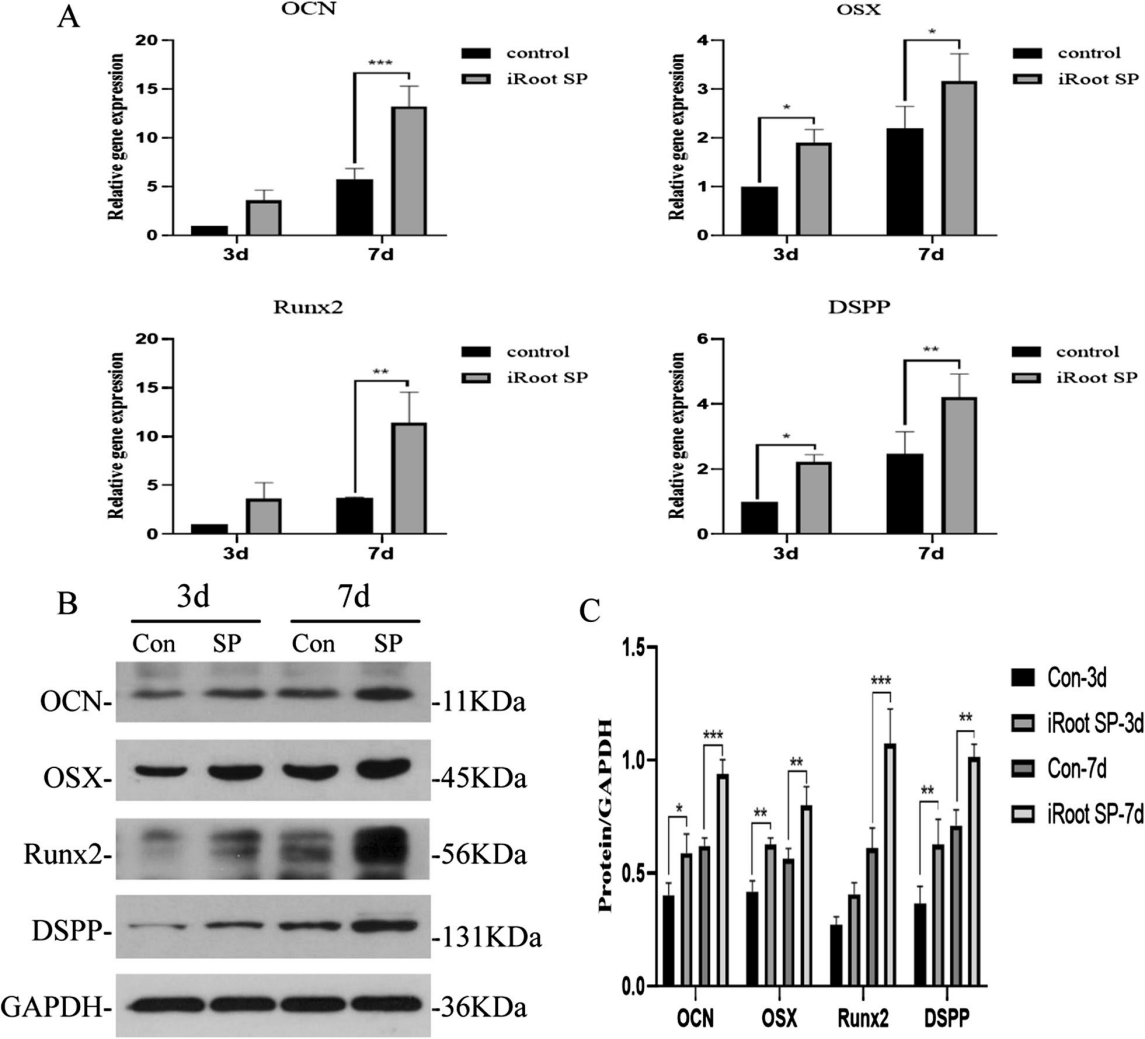
Effects of iRoot SP on osteogenic differentiation in hSCAPs. **A** Relative expression levels of the osteogenic genes OCN, OSX, Runx2, and DSPP in hSCAPs normalized to the housekeeping gene GAPDH. **B** Western blot results showing the upregulated protein levels of osteogenic markers in the 0.2 mg/mL iRoot SP group. GAPDH was used as a loading control. **C** Grayscale analyses of the results shown in (**B**). *P < 0.05, **P < 0.01, and ***P < 0.005. Error bars: mean ± standard deviation

To further determine the effect of iRoot SP on the osteogenic differentiation of SCAPs, cells were treated with 0.2 mg/mL iRoot SP. The qRT-PCR results show that the mRNA expression levels for the iRoot SP group are significantly elevated compared with the control group at day 7 (*P* < 0.05). However, no significant changes in the expression of Runx2 and OCN are detected at day 3 (Fig. 4A). Meanwhile, the relative protein expression levels of the osteogenic markers are consistent with those of the genes (*P* < 0.01) (Fig. 4B, Additional file 1: Figure S1 and Fig. 4C). Collectively, these data indicate that 0.2 mg/mL iRoot SP enhances the osteogenic differentiation of hSCAPs.

## Discussion

MSCs have wide application prospects in tissue-regenerative medicine. The hSCAPs used in this study are early MSCs present in developing apical dental papilla tissues that exhibit great potential for tissue regeneration owing to their proliferation activity and pluripotency [6, 9]. Moreover, it has been demonstrated that, compared with other odontogenic stem cells under the same conditions, hSCAPs present a higher mineralized-tissue-formation rate and thus show promise for the treatment of bone defects [22].

Numerous biochemical and biophysical factors guide stem-cell regeneration in different ways [7]. Nano-bioceramic root canal filling materials directly contact periapical tissue, isolating inflammatory environments and inhibiting microbial invasion, thus promoting the healing of damaged apical tissues [23]. Accordingly, the treatment of periapical diseases calls for root-canal-filling materials that provide appropriate microenvironments conducive to effective root-canal disinfection and the proliferation and differentiation of stem cells [19, 24].

iRoot SP is an injectable, pre-mixed bioceramic root-canal sealer with excellent sealing properties and antimicrobial activity [21]. Most studies on iRoot SP have focused on its feasibility as a root-canal-repair material. For instance, Zaki et al. found that the hydroxyapatite generated by iRoot SP can be used as a scaffold that accepts osteoblasts, and that bone tissue treated with iRoot SP reaches a state of almost complete healing within two months [25]. Thus, stem-cell therapies based on a combination of nanomaterials and stem-cell biology show promise for tissue repair in the clinic [26]. iRoot SP may be used as a potential regulator of hSCAP activity and osteogenic differentiation to improve the extracellular environment so as to better promote the regeneration and healing of periapical bone defects. Accordingly, on the basis of previous research results and preliminary data [27, 28], different concentrations of iRoot SP extract were selected for investigation in the present study.

Biomaterials for tissue engineering should exhibit excellent cytocompatibility and support cell growth and proliferation because cytotoxic biomaterials can cause cellular degeneration and delay wound healing [29]. In this study, the effects of iRoot SP on the proliferation of hSCAPs on the 1st, 3rd, and 5th day after treatment were evaluated by CCK-8 assays. The results showed that there are differences in the proliferation of hSCAPs treated with different concentrations of the extract. Lower concentrations of iRoot SP extract have no significant negative effects on cell proliferation, while the extract improves cell proliferation at 0.2 mg/mL.

Previous studies have demonstrated the relationship between cell biological behavior and calcium concentration [30]. An appropriate amount of calcium leached from the iRoot SP facilitates cell proliferation by activating mitochondrial matrix dehydrogenases [31]. However, iRoot SP at high concentrations inhibits the proliferation of hSCAPs. However, these findings are different from those reported by Zhang et al., who observed that iRoot SP has no cytotoxic effects after curing for 24 h [32]. The cytotoxic effects of iRoot SP may be explained by its high surface pH, which cause denaturation of adjacent cells and culture medium proteins. These inconsistencies may be due to the different setting time and stimulation modes applied for the test materials in our study [33].

Cell migration plays an important role in tissue regenerative and repair processes as it allows more cells to be recruited for repairing damaged sites [34]. In this study, wound-healing assays were used to detect the migration of hSCAPs treated with iRoot SP extract at three concentrations after 12 h. The results indicated that low concentrations promote cell migration, with the migration ability for the 0.2 mg/mL group being higher than those of the other groups. This result is in agreement with that of a previous study, which showed that iRoot BP Plus promotes dental pulp cell migration and pulp repair by activating FGFR-mediated signaling pathways, upregulating the expression of focal adhesion molecules and promoting stress-fiber assembly [35]. It has also been reported that the formation of a hydroxyapatite layer on the surface of a filling material promotes the recruitment of MSCs, leading to highly active tissue repair and/or regeneration [36].

As a functional marker enzyme, ALP is involved in the formation of mineralized tissues such as teeth and bone and is considered to be an early marker of osteogenic differentiation in odontogenic stem cells [37]. The results showed that 0.2 mg/mL iRoot SP extract significantly increases the ALP activity of hSCAPs, and Alizarin red staining and semi-quantitative CPC analysis also demonstrated that the calcium matrix produced by cells increases significantly under treatment at this optimal concentration. OCN, Runx2, OSX, DSPP are characteristic markers of osteogenic differentiation [37, 38]. Our results revealed that treatment of hSCAPs with 0.2 mg/mL iRoot SP extract creates a suitable microenvironment for periapical bone regeneration and upregulates the expression of osteogenesis-related genes and proteins. This result is consistent with previous findings indicating that iRoot SP upregulates the gene and protein expression of osteogenic-related factors in MG63 cells [18]. Chang et al. found that iRoot SP promote the osteogenic differentiation of periodontal ligament stromal cells (PDLSCs) by activating integrin receptors and downstream signaling molecules [20].

Related studies have demonstrated that the silicon and calcium ions released by iRoot SP during hydration and solidification may be the main active components of the material, and that their bioactivities affect cellular response [39]. The release of non-polar ions induces a series of biological reactions in MSCs, thus inducing tissue repair and regeneration [40]. It has been shown that MTA induces BMP-2 expression and calcification in human periodontal ligament cells through CaSR interactions, which are activated by the gradual release of calcium [41]. Another study demonstrated that silicon ions have a dual action, promoting osteoblasts and inhibiting osteoclasts [42]. The inorganic ions released by bioceramic materials have been identified as important signaling molecules and regulators that control numerous cellular activities that maintain the homeostasis of bones [43].

It should be noted that the present study has several limitations. First, the concentrations of the ionic products from iRoot SP were not determined. Furthermore, the signal transduction pathways of iRoot-SP-induced osteogenic differentiation in hSCAPs was not investigated. Additionally, osteogenic differentiation has a particular time profile, so longer observation periods may be needed to understand the fluctuation of the expression of osteogenesis-related factors during the complete process.

## Conclusions

iRoot SP is a promising calcium-silicate-based biocompatible material with potential application value in periapical bone regeneration. Our results demonstrate that iRoot SP extract at 0.2 mg/mL enhances hSCAP proliferation, migration, and osteogenic differentiation. Overall, this study provides valuable information for the further study of the mechanisms of bioceramic-mediated periapical bone repair.

## Supplementary Information

**Additional file 1**. **Figure S1**: Original gel images of Fig. 4B.

## Data Availability

The datasets used for the current study are available from the corresponding author on reasonable request.

## Abbreviation

hSCAPs: Human stem cells from apical papilla
MSCs: Mesenchymal stem cells
α-MEM: Alpha-modification of Eagle’s medium
FBS: Fetal bovine serum
MTA: Mineral trioxide aggregate
CCK-8: Cell counting kit-8
ALP: Alkaline phosphatase
OCN: Osteocalcin
OSX: Osterix
Runx2: Related nuclear facto 2
DSPP: Dentin sialo phosphoprotein
GAPDH: Glyceraldehyde 3-phosphate dehydrogenase
