# Supplementary figures and images for: Effects of iRoot SP on osteogenic differentiation of human stem cells from apical papilla
Source: BMC Oral Health. 2021 Aug 18;21:407. doi: 10.1186/s12903-021-01769-9 (PMC8371802; doi:10.1186/s12903-021-01769-9)

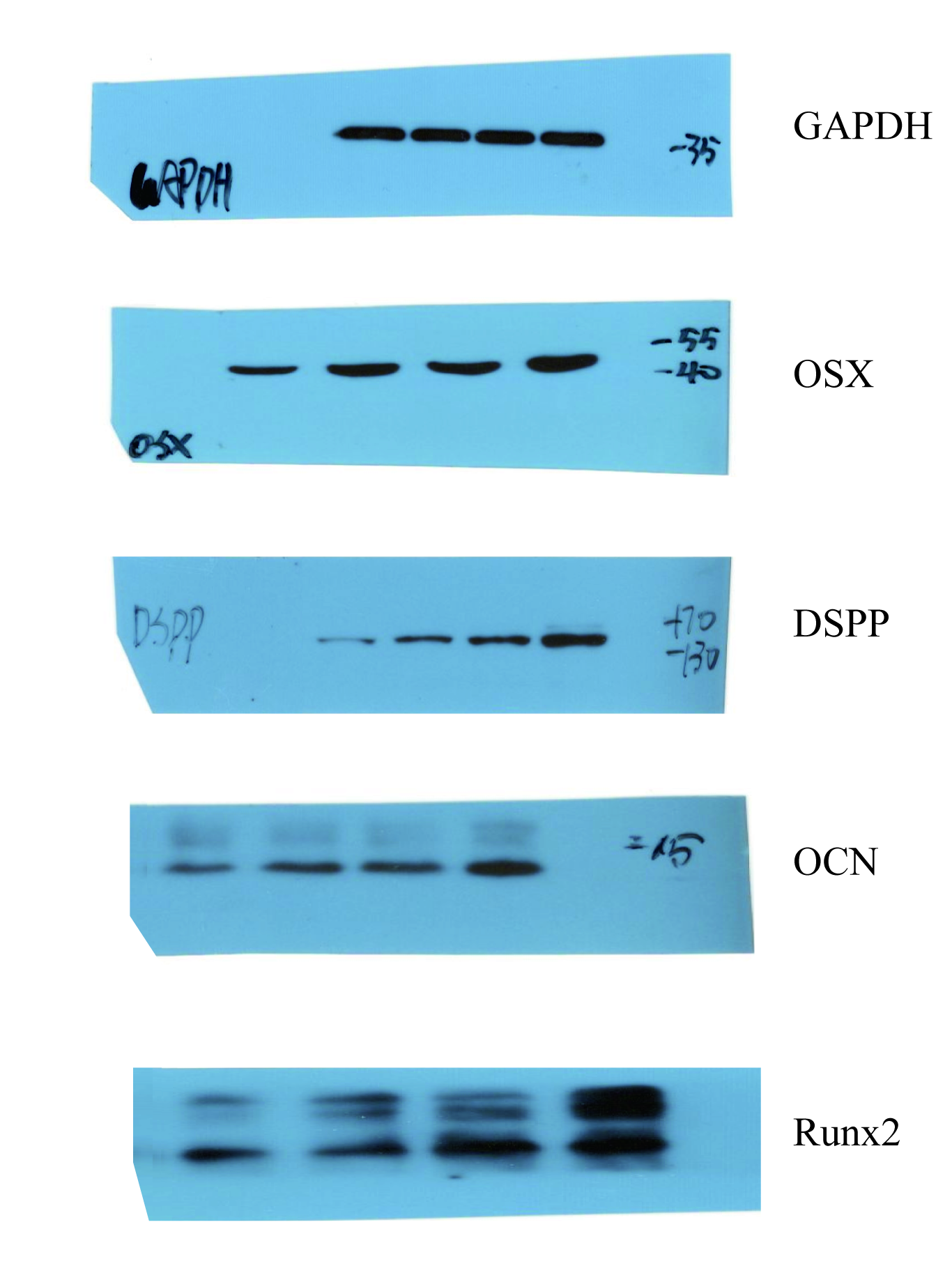

Supplement: Supplementary file 1 — Additional file 1. Figure S1: Original gel images of Fig. 4B. [file 12903_2021_1769_MOESM1_ESM.tif]
